# A systematic review of training methods to increase staff’s knowledge and implementation of positive behaviour support in residential and day settings for individuals with intellectual and developmental disabilities

**DOI:** 10.1177/17446295211022124

**Published:** 2021-07-05

**Authors:** Dearbaile Mahon, Edith Walsh, Jennifer Holloway, Helena Lydon

**Affiliations:** National University of Ireland, Galway, Ireland; Athlone Institute of Technology, Ireland; National University of Ireland, Galway, Ireland; National University of Ireland, Galway, Ireland

**Keywords:** intellectual and developmental disabilities, PBS, staff training, residential and day support services

## Abstract

Behaviour support plans (BSPs), if accurately implemented, have been found to increase skills and decrease challenging behaviour of individuals with intellectual and developmental disabilities. Training is essential for staff to acquire the skills necessary for accurate implementation. The aim of this systematic literature review was to evaluate procedures used to train staff in Positive Behaviour Support (PBS), on both knowledge of PBS and implementation of BSPs. Systematic searches of 4 databases identified 18 studies as meeting criteria. Findings indicate that description alone was not consistently effective in increasing knowledge and should be used in combination with other training strategies. Staff’s implementation of BSPs were increased by different combinations of the following training components: description, feedback, modelling, role-play, monitory incentive, and escape contingency. To identify evidenced based practice when training staff on BSPs, it is necessary to evaluate active and feasible training components from current training models.

## Introduction

It is estimated that 10–15% of people with intellectual and developmental disabilities present with challenging behaviour (CB; Allen et al., 2005; Emerson et al., 2001). Challenging behaviour negatively impacts on an individual’s educational and social life and if not addressed may persist across an individual’s lifetime (Kiernan and Kiernan, 1994). This can result in limited access to the community, education, employment and social relationships (social isolation, exclusion) (Emerson et al., 2001; Horner et al., 2002; Royal College of Psychiatrists, 2007). Therefore, it is crucial to identify suitable interventions to increase the quality of life of individuals and those supporting them, as CB can impact on staff working directly with individuals engaging in such behaviours, often resulting in staff burn out and high staff turnover (Devereux et al., 2009; Felce et al., 1993).

Positive Behaviour Support (PBS) is one such approach which has yielded positive outcomes in increasing positive skills and reducing CB for individuals with intellectual and developmental disabilities, thus, increasing overall quality of life (LaVigna and Willis, 2012). PBS evolved from applied behaviour analysis in the 1980s and 1990s. Since this time, there have been developments in the core understanding of PBS as a value based person focused approach, using long-term, systems change and educational methods to achieve outcomes (Carr et al., 2002). PBS is now recommended as best practice within special educational law in the USA (IDEA, 2004) and is mandated within residential services in Ireland for individuals who engage in CB (Health Act, 2007) in the form of a Behaviour Support Plan (BSP). This has paved the way for PBS to be adapted both at an organizational and individual level, within services.

A BSP incorporates several behavioural interventions across four main categories: i) environmental accommodations (ecological; to reduce the likelihood of CB occurring); ii) skills teaching interventions (positive programming; teaching alternative appropriate skills to replace CB); iii) direct interventions (focused support strategies; alternative appropriate skills are reinforced over CB); iv) and reactive strategies (non-aversive strategies to ensure safety and the dignity of the person when responding to CB) (LaVigna and Willis, 2005).

For a BSP to have the desired effect of reducing CB and improving the quality of life of individuals with intellectual and developmental disabilities, accurate implementation is essential. Frontline staff often hold primary responsibility for implementation of BSPs, therefore, staff training is pivotal in achieving accurate implementation (Hastings and Brown, 2000; Reid, 2004). Staff training is also vital from an ethical perspective, as incorrectly implemented interventions can impact negatively on both individuals supported by the service (who will be referred to as clients) (Carroll et al., 2013), and staff (Kazemi et al., 2015). When contemplating staff training it is important to consider the content, the process of delivery, and the resulting outcomes (e.g. skills acquired by staff). Given that PBS is mandated through legislation, this has led to system change within organizations, which includes providing training on PBS, which was not evident to the same degree when PBS was coming to the fore in the 90s. Existing literature on staff training indicates that, a variety of different training packages (e.g. instructions, modelling, role-play, and feedback) have been used for teaching behavioural intervention.

A large-scale scoping review of training practices for staff supporting individuals with intellectual and developmental disabilities indicated that staff implementation was the most frequently evaluated variable, however few studies focused on client outcomes (Gormley et al., 2019b). A meta-analysis examining the impact of training staff that support individuals with intellectual disabilities and engage in CB indicated that while training was moderately effective in changing staff outcomes, there wasn’t conclusive evidence of change in levels of CB for the supported individuals (Knotter et al., 2018). To date, MacDonald and McGill (2013) is the only Systematic Literature Reviews (SLR) undertaken to examine the effectiveness of staff training for interventions targeting CB of individuals with intellectual and developmental disabilities specifically for PBS.

MacDonald and McGill (2013), examined the effects of staff training in PBS on outcomes of staff, including knowledge or skills. The SLR reported on the length, format and content of the training, however, insufficient detail was provided on the methods used to train staff, preventing the training components responsible for training efficacy to be identified. A recent review by Brady et al. (2019) undertook a SLR which summarized outcomes of studies with respect to procedural fidelity of behavioural interventions (which included but was not specific to BSPs) for individuals with intellectual and developmental disabilities in human services. They concluded that feedback was the most commonly used training strategy, with moderate effect sizes when used in isolation and large effect sizes when used as an element of a training package. Whereas instruction and teaching had the highest effect size but were only ever used in combination with other training strategies. This review was completed across human services settings, where there are large variances in available resources, with a wide range of participants and included studies which were training both BSPs and single behavioural interventions.

Consequently, the objective of the current systematic literature review is to extend on the review undertaken by MacDonald and McGill (2013) to evaluate procedures of training staff in PBS from 1990 to 2019 across children and adults with intellectual and developmental disabilities on the outcome variables of knowledge and implementation. In addition, the current review aims to refine the review carried out by Brady et al. (2019) to examine the effects of staff training on the implementation of BSPs in residential and day settings for individuals with intellectual and developmental disabilities.

## Methods

### Protocol and registration

Preferred Reporting Items for Systematic Reviews and Meta-Analyses guidelines (Moher et al., 2009) were adhered to throughout the SLR process. The SLR protocol was developed by the research team and was registered on the International Prospective Register of Systematic Reviews (PROSPERO) database: http://www.crd.york.ac.uk/PROSPERO/display_record.php?ID=CRD42017081748.

### Search strategy

Four electronic databases were searched separately: Psych INFO, Scopus, Psychology and Behavioral Sciences, and Web of Science; as well as hand searches of reference lists of identified studies. The key terms were separated into Lists; List 1: staff train*, carer train*, paraprofessional train*, List 2: positive behav*, behavior* intervention, List 3: intellectual disab*, developmental disab*. The key terms were entered so that every term from List 1 was paired with every term from List 2 and from List 3. The search was limited to peer reviewed journals, with English language, from 1990 to July 2019.

### Search procedures

**Figure 1.**
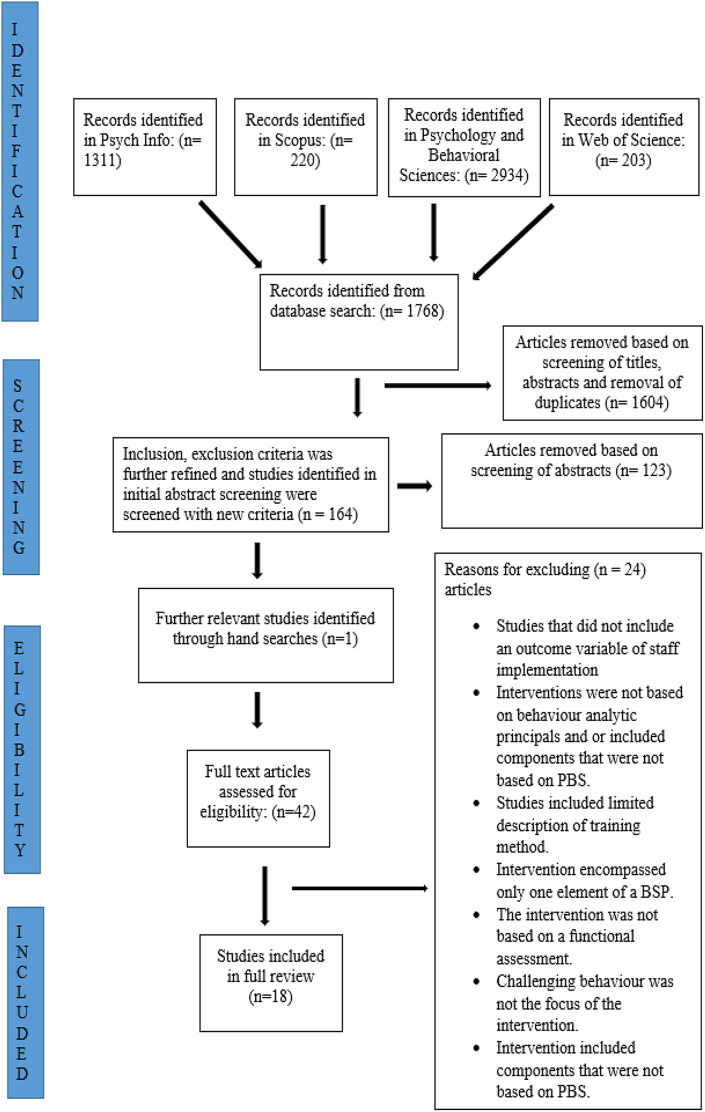
Summary of search strategies.

Systematic searches were undertaken in two stages. Stage 1 consisted of screening titles and abstracts according to the inclusion and exclusion criteria. Ambiguous abstracts were further reviewed in Stage 2 which consisted of using the inclusion and exclusion criteria to review full text articles, identifying studies to be included in the SLR. See Figure 1 for a summary of search strategies.

### Inclusion and exclusion criteria

Horner et al. (1990) highlight nine characteristics of PBS:

an emphasis on lifestyle change, functional analysis, multicomponent interventions, manipulation of ecological and setting events, emphasis on antecedent manipulations, teaching adaptive behaviour, building environments with effective consequences, minimizing the use of punishers, distinguishing emergency procedures from proactive programming, and social validation and the role of dignity in behavioural support. (pp. 127–129)

This definition was used to inform the inclusion/exclusion criteria of the current review.

Studies were included if: a) They had a quasi or experimental design; b) Staff participants supporting individuals with intellectual and developmental disabilities in residential and day settings; c) Training provided on behavioural principles and/or BSPs (including behavioural interventions from two or more of the four categories of multicomponent BSPs: environmental accommodations, skills teaching interventions, direct interventions and reactive strategies); and the methods of training staff were clearly outlined (e.g. feedback, role-play); d) Data on staff implementation of BSPs to reduce CB or staff knowledge about behavioural principles was included as a dependent variable post training. Horner et al. (1990) highlighted ‘minimizing the use of punishers’ not eliminating the use of punishers as a characteristic, for this reason comprehensive BSPs which incorporate a punishment element were included in the current review.

Studies were excluded if: a) They had qualitative data or a review research design; b) The participants trained were caregivers or worked outside residential or day settings such as schools; and c) No description was provided on staff training components; if the intervention was not informed by functional assessment, solely focused on training data collection methods, encompassed only one element of a BSP or included components that were not based on PBS (training on therapies with a behavioural component, e.g. dialectical behaviour therapy, cognitive behavioural therapy, acceptance and commitment therapy, mindfulness-based PBS); d) CB was not the focus of the intervention; if staff perception/staff attitudes of CB/PBS or staff efficacy/confidence were the sole dependent variables.

### Data extraction

Articles identified as meeting the inclusion criteria were summarized and data was extracted for: a) reference and the country where the research was undertaken; b) participants information (i.e., number included, setting, previous experience with PBS); c) training content; d) training components (i.e., description, feedback, modelling, rehearsal); e) format/duration; f) research design; g) dependent variables/measures; and h) staff and client outcomes.

#### Training components operational definitions

Instruction included both written and vocal, sessions where PBS theory, including functions of challenging behaviour or information on the behavioural interventions were the focus. Discussions were included within this. Modelling included the trainer providing a model of how to implement the intervention with a confederate or with client either *in vivo* or a video format outlining correct and incorrect examples. Rehearsal included the trainee role-playing the intervention with a confederate, colleague or carrying out the intervention with the client. Feedback included vocal or written feedback from trainer both corrective feedback for partial or incorrect implementation and praise for elements correctly implemented. Feedback included immediate, delayed, *in vivo*, via videos. Fluency training involved the use of a precision teaching approach to train to fluency using SAFMEDS. Monetary incentive included if staff met a set criterion, they received financial payment. Negative reinforcement included if the staff member achieved a set criterion during role-play or *in vivo*, the session ended versus if they didn’t meet the criterion they continued rehearsing until the criteria was met.

##### Certainty of evidence

Certainty of evidence was used to describe the methodological rigour of studies and was rated as ‘suggestive, preponderant’ or ‘conclusive’ (Smith, 1981). Studies were described as suggestive if they did not utilize a true experimental design (e.g., if they used a quasi-experimental design) to evaluate outcomes. Studies were described as preponderant if they had: (i) an experimental design (e.g., multiple-baseline design/between groups with random assignment); (ii) dependent variable’s that were operationally defined; (iii) inter-observer agreement for at least 20% of sessions with agreement at 80% or higher; (iv) interventions were explained clearly enough to enable replication; (v) or were not able to control for other variables that could have impacted on intervention outcomes. Conclusive was the highest level of certainty. Studies were rated at this level if they contained all the preponderant level characteristics but included treatment fidelity measures. Also, if they attempted to control for other variables/factors that could have impacted on intervention outcomes.

##### Synthesis of results

A descriptive narrative synthesis was used to summarize the evidence relating to the effectiveness of different types of staff training in PBS/BSP. Staff training results, were described as positive, negative, or mixed (e.g., Machalicek et al., 2008). Outcomes for single subject research design studies were described as positive if visual analysis indicated improvement for all participants on implementation. Mixed, if visual analysis indicated improvement for only some of the participants on implementation. Negative, if visual analysis indicated no improvement for any participants on implementation. Outcomes for between subjects’ design and within subjects design studies were described as positive, if there were statistically significant improvement for participants (PBS group and pre-post) on all dependent variables related to PBS knowledge and implementation. Mixed, if there were statistically significant improvements for participants (PBS group and pre-post) on some but not all dependent variables related to PBS knowledge and implementation. Negative, if there were no statistically significant improvements for participants (PBS group and pre-post) on any dependent variables related to PBS knowledge and implementation. Client outcomes were described using the same classification.

### Reliability of search procedures and inter-rater agreement (IRA)

Stage 1 and Stage 2 search strategies were conducted independently by the first and second author with 94% and 89% agreement respectively. To calculate the inter-rater score, the number of agreements was divided by the number of disagreements, then added with agreements and multiplied by 100%. A third rater (fourth author) was used in the event of all disagreements and group discussion was used to reach consensus on inconsistencies until 100% agreement was reached. The first author undertook data extraction and the second author independently performed inter-rater agreement on each variable extracted (including certainty of evidence and synthesis of results) with 88% agreement. In the event of all disagreements consensus was reached through discussion until 100% agreement was reached.

## Results

Eighteen articles met the criteria for inclusion in this review from 1990 to 2019, 1 from Australia, 3 from Ireland, 7 from the UK, 6 from the USA, and 1 from the Netherlands. Included articles are summarized in Table 1 in alphabetical order. Table 2 summarizes the staff training methods utilized for each study and the associated intervention outcomes.

**Table 1.** Summary of identified studies.

Reference (country)	Participants and Participant Experience	Training Content	Training Components	Format / Duration	Design & Measures	Staff (S) & Client (C) Outcomes & Certainty of evidence:
*Knowledge*						
**Berryman, et al. (1994)** (USA)	83 residential staff supporting indiv’s with DD and psychiatric disorders **PBS Experience**: Initially, 2 weeks of training in traditional behav. management techniques 2 days of training yearly	**Positive Behavioural** **Interventions:** – FA – Viewing clients as similar to indiv’s without a disability – Treating clients with dignity and respect – Data based decision making Non-aversive training focused on FCT Traditional training focused on R+	D: W/ discussion	**Format:** Group **Duration**: 1-day workshops	**Design**: WSD & BGD: randomly assigned to either non-aversive or traditional behaviour management strategies **Measures**: 1. Attitudes Towards Disabled Persons Scale- Form A 2. CACBS	**S Outcome**: Participants in the non-aversive group (experimental) had statistically significant differences in scores on the CACBS, post training compared to the traditional group (control). **Certainty of evidence**: Preponderant due to lack of TF
**Branch et al. (2018)** (UK)	24 staff (5 staff teams) working with indiv’s with DD and severe CB with no previous knowledge of precision teaching or fluency training **PBS Experience**: Level of knowledge of PBS NR	BSPs **Concepts/strategies**: – Description/function of CB – PS – FCT – RS	**D:** W/ discussion on BSPs **Consultation as usual**: **D:** Written description of BSP to learn **Fluency training (FT)**: **D:** BSP (flashcard format) **M:** A fluency training practice session **R:** Practice 3 times daily for 1 minute until fluency aim reached and maintained across 3 successive attempts. Practice drill for a pack of flashcards they had reached their fluency aim on, once a week	**Format**: Group (NR) **Duration**: All staff: 3-hour workshop FT Group: 1-hour instruction for fluency training 4-week period for both groups	**Design**: Quasi-experimental design **Measures:** 1. Test of component skills (30 flashcards from Pack 1 and 30 from Pack 2 individualized for their client’s BSP): pre- and post and at 6 weeks from baseline 2. A written composite test completed: 4 and 6 weeks from baseline	**S Outcome**: Control had negative outcome, experimental group had a positive outcome: The FT group obtained statistically significant gains in component skill recall and achieved higher composite test scores **Certainty of evidence**: Suggestive due to a quasi-experimental design
**Campbell and Hogg (2008)** (UK)	276 staff supporting people with ID and CB in a variety of service settings **PBS Experience**: Level of prior knowledge of PBS NR	**Group 1**: Course ‘Approaches to People with CB’ Outlined: – What is CB? – Role of Staff – Constructional Approach – Behavioural Principles, – Aversive & Non-Aversive Approaches, – Behavioural Observation – Changing Behaviour Settings – Quality in a Behavioural Approach **Group 2**: Course ‘Approaches to Sexual Abuse of Adults with Learning Disabilities’ **Group 3:** no accredited course undertaken	Individual activities, case studies, up to date research findings, structured text and current references	**Format:** Individual: Distance learning **Duration**: Each module was expected to be complete within a 3-month period	**Design**: WSD & BGD: 3 groups: 1 experimental, 2 controls. **Measure**: CBRQ Administered at 4-time points: 3 months, prior to training, just prior to training, immediately following training and 3 months post training	**S Outcome**: Mixed for both groups as the experimental group outperformed the two control groups, on two of the five-dimension measures, Cause and Treatment/Control **Certainty of evidence**: Suggestive as although it used both a within and between groups design, the indiv’s were not randomly assigned to the groups, IOA or TF were NR
**Dowey et al. (2007)** (UK)	54 direct care staff supporting indiv’s with ID **PBS Experience**: Level of prior knowledge of PBS NR	– Service values & QOL issues for people with ID – Intro to ABA – Role of the environment with CB	**D:** Lectures Handouts & small group exercises RP	**Format**: Group **Duration**: Single day workshop	**Design**: WSD **Measure**: SIBUQ	**S Outcome**: Positive, the there was a significant change in behavioural correct explanation. **Certainty of evidence**: Suggestive due to a quasi-experimental design
**Hardesty et al. (2014)** (USA)	101 new employees of an inpatient hospital unit supporting indiv’s (ages 3–21 years) with ID who exhibited severe CB **PBS Experience**: 2.5-weeklong orientation consisting of in-service trainings: basic behavioural principles, behavioural treatment technique, behavioural skills training strategies	Basic behavioural principles	**D:** W/discussion **F:** Both individual and to the group depending on condition	**Format**: Group **Duration**: 2 hr sessions	**Design**: BGD randomly to either response card or standard training. **Measure**: 1. Oral quizzes (10 questions): during training. 2. Written Quiz: 1–2 days after training 2 weeks after training. 3. Social validity survey (response card group) 4. Participant on task beh. 5. Accurate responses	**S Outcome**: Control, mixed, experimental group positive, as while the response group was significantly more accurate than the standard group, in the in-service training and for Quiz 2, groups were comparable for accuracy in Quiz 1 **Certainty of evidence**: Suggestive as not enough detail given for replication, could not access main measure used, TF NR
**Lowe et al. (2007)** (UK)	275 staff, nursing assistants (non-registered staff) nurses & managers (registered staff) in specialist health care service **PBS Experience**: Level of knowledge of PBS prior NR	**PBS training**: – Identify service mission – Promote fundamentals of care – Contribute to personal centred planning – Defining cb – 3-stage intervention model – Active support – Community profiling – Contribute to PSR – Supervision of support – Foundations of Communication	Taught course: **D:** Lectures, Video tapes Individual and group work Practical exercises and group discussions Course book containing all the taught material. Followed by Individual, in situ, practical instruction	**Format**: Group **Duration**: 80 hrs direct teaching across 10 consecutive days 32 hrs home-based study leave 40 hrs work-based study time Expected to contribute 28 hrs of personal time	D**esign**: WSD & BGD **Measures** Pre-and-post 1. ERCB 2. Challenging Behaviour Staff Perceptions Questionnaire Self efficacy Scale 3. Confidence in Coping with Patient Aggression Instrument 4. Fifteen sets of questions on knowledge selected from across the assessment portfolio for the 10 course units. 5. Challenging Behaviour Attribution Scale	**S Outcome**: Mixed for both groups, as although, there were significant increases in knowledge, this reverted to baseline levels at a 1 year follow-up **Certainty of evidence**: Suggestive as randomization to groups was not employed and TF NR
**McGill et al. (2007)** (UK)	79 students on the Diploma cohorts from 1998-2000 **PBS Experience**: 54 had limited experience with CB training (not specified)	**First year focus**: – Social role valorization – ABA – Observation – Communication – Participation – Teaching – Implementation **Second year focus**: – FA & Intervention	Taught in workshops (NR) Competency-based with most of the assessed work being reports or videos of practical work with service users	**Format: Mixed (NR)** **Duration**: Series of 2–4-day workshops over the course of 2 years part time First year 29 days. Second Year 28 days Practical work in their own agencies	**Design**: WSD **Measures**: 1. SIQUB 2. CHABA 3. Vignettes on behavioural function 4. ERCB	**S Outcome**: Mixed: Knowledge increased significantly on the Causal Behaviour Correct scores and Knowledge Behaviourally Correct scores on the SIBUQ and on the CHABA, but on the Vignettes on behavioural function a significant difference was not found for the escape vignette post training. **Certainty of evidence**: Suggestive due to a quasi-experimental design
**Rose et al. (2014)** (UK)	65 residential care staff who supported indiv’s with ID **PBS Experience**: Required to not have attended a challenging behaviour training within the last 6 months.	**CB course**: – Attitude – Behaviour – Observation – Understanding – Techniques	**D**: PowerPoint slide, Individual, small group exercises and guided discussion Detailed description of the course	**Format: Group** **Duration**: One-day course	**Design**: WSD **Measures**: 1. Controllability Beliefs Scale 2. Pre- and Post training & 2 months post. 3. The Five-Minute Survey	**S Outcome**: Positive, statistically significant changes in staff attributions after training **Certainty of evidence**: Suggestive due to a quasi-experimental design
**Tierney et al. (2007)** (Ireland)	48 staff from ID organizations in the Health Service Executive **PBS Experience**: Level of knowledge of PBS prior NR	**Day 1**: PBS **Day 2**: Attitudes Crisis Prevention Institute Non-Violent Crisis Intervention Training Programme’. **Day 3:** Stress & it’s impact	**D**: Theoretical presentation, w/discussion. Group work **RP** Practical skills Teaching	**Format**: Group **Duration**: 3 days 5 training courses over 1 year, approximately 14 staff attended each course.	**Design**: WSD **Measures**: 1. CHABA 2. Self-efficacy scale 3. ERCB	**S Outcome**: Negative, no significant changes in causal beliefs about CB **Certainty of evidence**: Suggestive due to a quasi-experimental design
**van Oorsouw et al. (2010)** (Netherlands)	70 direct care staff supporting indiv’s with ID in residential homes and day-care centres **PBS Experience**: No participation in a comparable training regarding the management of CB for at least 2 years	**Theory**: – Causes & topography of CB & escalation signs, recognizing the symptoms of trauma & response needed – Physical intervention skills	Small group exercises Guided discussions **RP**	**Format**: Group **Duration**: 7 sessions of 3½ hrs concerning the theory of CB 5 sessions of 1½ hrs concerning physical intervention training.	**Design**: Quasi-experimental pre & post-test control group design **Measures**: 1. Knowledge questionnaire 2. Observation manual for the assessment of the staff physical intervention skills 3. Social validity measure	**S Outcome**: Positive: significant increase in knowledge of CB, at follow-up while the scores were significantly larger than pre-test scores, they were significantly smaller than post-test scores **Certainty of evidence**: Suggestive due to a quasi-experimental design
*Knowledge and Implementation*						
**Gormley et al. (2019a)** (Ireland)	104 frontline staff supporting people with ID **PBS Experience**: Intervention participants: 75.9%, 87%, 85.2% and 79.6% sequentially reported no previous training in reinforcement, systematic prompting, FCT and task analysis. Control participants: 75.9%, 87%, 85.2% 48.9%, 71.1%, 68.9% and 66.7% sequentially reported no previous training in reinforcement, systematic prompting, FCT and task analysis.	**Training modules**: – R+ – Systematic prompting – FCT, Task analysis	**D**: via PowerPoint presentation with accompanying manual **D**: embedded in general case video M **R**: with confederate and **F**: individually Developed communication plan for SU they were supporting.	**Format**: Group **Duration**: Across 4 months, 20 hours across 3 consecutive days. Mastery criterion: 90% correct implementation across 3 consecutive observations	**Design**: Parallel cluster RCT, with control. WSD – intervention group on knowledge measure **Measure**: 1. Multiple choice questionnaire on knowledge of each module 2. Test of Knowledge 3. Maslach Burnout Inventory 4. Minnesota Satisfaction Questionnaire 5. Occupational Self-Efficacy Scale 6. The Shortened Ways of Coping Questionnaire 7. Percentage correct of implementation of each skill 8. Observations of FCT implementation with client 4 weeks after training 9. Training Acceptability Survey 10. Attitudes to Evidence-Based Practice Questionnaire 11. Perceptions of Supervisory Support Scale	**S Outcome**: Mixed: Between group and within subjects knowledge scores for intervention group were significantly higher post intervention Within subject skills: NI (No baseline) **Certainty of evidence**: Preponderant due to no baseline data being taken for skills
**MacDonald et al. (2018)** (Uk)	72 first level managers in a community-based social care provider and the staff they manage **PBS Experience**: Level of knowledge of PBS NR Clients: 72 indiv’s with ID the managers support	Intro to PBS **Functional Assessment**: – Defining and recording Behaviour – Functions **Behaviour support planning**: – Activity and PSR – interpersonal – new skill – focused support – reactive	Workshops and between workshop activities **RP** **F**	**Format**: Group 10 days of workshops. Part of PSR, not clear	**Design**: Non-randomized control group study with both BGD and WGD. **Measures**: 1. Multiple choice questionnaire on knowledge 2. PSR 3. The Aberrant Behaviour Checklist 4. The Active Support Measure 5. The Adaptive Behaviour Scale 6. Behaviours Recording Forms 7. CHABA 8. The Guernsey Community Participation and Leisure Assessment 9. Momentary Time Sampling: For quality of staff support and SU engagement 10. Practice Leadership Questionnaire	**S Outcome**: Mixed: Significant difference in knowledge tests for managers but no significant difference on the CHABA post intervention PSR, no baseline measure pre-training **C Outcome**: Mixed Significant reductions in CB, no significant changes to QOL **Certainty of evidence**: Suggestive due to non-randomized and lack of TF
**Macurik et al. (2008)** (USA)	38 residential direct support staff supporting three selected indiv’s with severe disabilities **PBS Experience**: Level of knowledge of PBS prior NR	**Individualized Intervention Plans**: – PS – FCT – DR – Consequences for CB	Live Training: **D:** Written summary, vocal description and sought questions Video training: **D:** BA describing each component with visual bullet points on screen	**Format**: Group **Duration**: Live training averaged 31, 23, and 46 min Video training averaged 21, 10, and 35 min.	**Design**: BGD **Measures**: 1. Written knowledge quiz. 2. Checklist of accuracy of components of BSP accurately implemented 3. Duration of training	**S Outcome**: NI, examined the statistical difference between providing a description in vivo or by video rather than the statistical significance of an increase in knowledge pre- and posttraining **Certainty of evidence:** Conclusive, due to a between groups design with random assignment, IOA, TF reported
*Implementation*						
**Courtemanche et al. (2014)** (USA)	2 care staff and 1 paraprofessional supporting 3 indiv’s with varying diagnosis who engaged in SIB. **PBS Experience**: Level of knowledge of PBS prior NR	**BIP’s:** – DR – FCT – RB – EE	**MI**: Across all phases for correct implementation or higher scores on quiz **Phase 1** **D**: Written with explanation **D:** Written with M and RP **Phase 2** (in vivo training) **F:** paired with MI, and an escape contingency **Phase 3** Integrity Intervention (Video review with both F and MI) **Phase 4** Frequency that F & MI were delivered was decreased.	**Format**: Individual **Duration**: NR Criteria: 100% correct implementation of the BIP for first RP of the session for 3 consecutive sessions, across 3 days 10 min observation if accurately implemented with client 10 min for Dyad 1 and Dyad 3	**Design**: MBD across dyads **Measure**: 1. Staff Performance Score: Total amount of points earned ÷ by the total number of applicable steps of the plan (× 2) × 100. 2. Frequency of client SIB 3. Social validity survey	**S Outcomes**: Positive across all phases, with high staff performance scores, meeting criteria. In the trainer’s absence performance scores were lower, in Phase 1 and Phase 2 **C Outcomes**: Phases 2 and 3 were positive as SIB was below baseline levels across all clients. Phases, 1 and 3 were mixed, as SIB levels were variable across 2 clients **Certainty of evidence**: Conclusive, due to an experimental MBD across dyads, IOA, TF reported.
**Crates and Spicer, (2012)** (Australia)	32 staff working for Disability Services Tasmania and the 32 indiv’s they support **PBS Experience**: Level of knowledge of PBS prior NR	**Level 1 training**: – FBA, FCT, PS, RS and emergency management within a non-aversive framework **Level 2 training**: – Longitudinal practicum, comprehensive functional assessment, developing a multi-element BSP and implementation	**D**: Lectures, Socratic discourse, -Reading assignments -Practicum assignments. Repeated practice Group activities **F:** Both individual written & group **M**	**Format**: Group (NR) **Duration**: Level One: 4 days, 6 hours a day Level Two training: longitudinal practicum, involving 9 days spread over a period of 9 months (repeated in the years 2006 to 2009)	**Design**: Repeated measure design **Measures**: 1. PSR at 3 months 2. Assessment and Intervention Plan Evaluation Instrument 3. Occurrence of target behaviours Episodic severity (ES) 4. Social validity survey	**S Outcome**: NI, reported PSR at only one-time point PSR results for 23 reports, the mean PSR score at 3 months was 47% (range 19–86%) **C Outcome**: Positive, significant improvements, target behaviour in 27/30 cases showed a reduction in ES at a follow-up of 3-months and a significant reduction in occurrence **Certainty of evidence**: Suggestive due to a quasi-experimental design
**Jarmolowicz et al. (2008)** (USA)	40 staff from an inpatient unit for indiv’s with DD who engaged in severe cb 20 staff with at least 1 year of graduate study in ABA 20 direct care staff with no formal training in ABA	– FCT & Extinction	**D**: Technical or non-technical written description of procedures **RP**: W/confederate	**Format**: Individual **Duration**: NR A single 40-trial session with a confederate who played the role of a client.	**Design**: BGD **Measure**: Percentage correct of implementation Likert type scale Questionnaire	**S Outcome**: Control: negative, experimental group: positive with statistically higher integrity for the non-technical than the technical group **Certainty of evidence**: Preponderant due to TF NR
**McClean and Grey (2012)** (Ireland)	61 staff: 55 frontline staff, 2 intensive support workers, one behaviour therapist, and 3 clinical psychologists **PBS Experience**: Level of knowledge of PBS prior for frontline staff **NR** **Clients**: 49 adults with ID, 12 children defined as under 18.	**BSPs** – EA – ST – Direct Interventions – RS	Person Focused Training **F**: Written and verbal at each stage Background assessment Functional assessment Intervention design Implementation	**Format**: Group & Individual (NR) **Duration**: 5-year period. 10 full days of training & coaching across 9 months	**Design**: Repeated Measures Design **Measure**: 1. PSR. 2. Baseline, 6 month and follow-up ratings for frequency, management difficulty, and episodic severity of the target behaviours 3. Real-time behaviour recording 4. The Challenging Behaviour Rating Scales 5. Treatment Acceptability Rating Form–Revised	**S Outcome**: NI, reported PSR at only one-time point Mean implementation rate of 67.7% **Certainty of evidence**: Suggestive due to a quasi-experimental design
**Shore et al. (1995)** (USA)	Direct-care staff members supporting indiv’s with varying diagnosis **PBS Experience**: Level of knowledge of PBS prior NR Clients: 8 indiv’s with varying diagnosis who engaged in SIB	– NCR, escape extinction, restraint fading	**Baseline 2** **D:** Review of written treatment procedure & description. **M:** Videotape showing implementation **Training:** **Part 1** data collection and calculation training, review of treatment procedure Criteria, 100% reliability with supervisor **Part 2** implementation training: **F:** from direct work with client Criteria: Independent implementation of procedure with 100% accuracy. **F:** twice weekly from supervisor	**Baseline 2**: **Format**: Group Training: **Format**: Individual (NR) **Duration**: NR	**Design**: MBD & Multiple Probe Design **Measures**: 1. Implementation calculated by dividing the number of correct staff responses by the number of observed opportunities. 2. Clients problem behaviours, and compliance	**S Outcome:** Baseline 2, negative: variable responses across all variables. Post training, positive: Increased correct implementation across antecedent, reinforcement and consequence responses **C Outcome**: Baseline, mixed: increase in levels of compliance from low to moderate levels for one student. Post training, mixed: improvements from baseline levels across all participants, there was a variable increase for one student’s inappropriate behaviour. **Certainty of evidence**: Preponderant due to not clear if TF was reported & not enough detail on data collection training

Note* ABA = applied behavioural analysis, BA = behaviour analyst, *Behav = behaviour, BGD = between groups design*, BIP = behaviour intervention plan, *BSP = behaviour support plans*, C = client, CACBS = causal attributions for challenging behaviour scale, *CB = challenging behaviour*, CBRQ = challenging behaviour representation questionnaire, CHABA = challenging behaviour attribution scale, *D = description, DD = developmental disability, DR = differential reinforcement, EA = environmental accommodation, EE = environmental enrichment*, ERCB = emotional reactions to challenging behaviour scale (extended version), *F = feedback, M = modelling, FA = functional analysis*, FBA = functional behaviour assessment, *FCT = functional communication training*, GW = group work, Hrs: hours, ID = intellectual disability, *Indiv’s = individuals*, IOA = inter-observer agreement, *MBD = multiple-baseline design*, MI = monetary incentive, *NCR = non-contingent reinforcement, NI = not interpretable, NR = not reported, PBS = positive behaviour support, PS = preventative strategies, PSR = periodic service review*, QOL = quality of life, *r+ = positive reinforcement, R = rehearsal, RB = response blocking*, RCT = randomized control trial, *RP = role-play, RS = reactive strategies*, S = staff, *SE = setting events, SIB = self-injurious behaviour*, SIBUQ = modified self-injury questionnaire, *ST = Skills teaching*, SU = service user, *TF = treatment fidelity*, W/ = with, *WSD = within subjects design.*

**Table 2.** Summary of training components, outcomes and certainty of evidence.

Reference		Description	Fluency Training	Modelling	Role-play	Feedback	Escape Contingency	Monitory Incentive	Staff Outcomes	Client Outcomes	Certainty of Evidence
*Knowledge*											
**Berryman et al. (1994)**	Traditional	x	-	-	-	-	-	-	N (C)	N/A	P
	Non-Aversive	x	-	-	-	-	-	-	Pos **(I)**		
**Branch et al. (2018**)	Consultation as usual	x	-	-	-	-	-	-	N **(C)**	N/A	S
	Fluency	x	x	-	-	-	-	-	Pos (I)	N/A	
**Campbell and Hogg (2008)**		x	-	-	-	-	-	-	M	N/A	S
**Dowey et al. (2007)**		x	-	-	x	-	-	-	Pos	N/A	S
**Hardesty et al. (2014)**	Standard Group	x	-	-	-	x	-	-	M **(C)**	N/A	S
	Response Card Group	x	-	-	-	x	-	-	Pos **(I)**	N/A	
**Lowe et al. (2007)**		x	-	-	-	-	-	-	M	N/A	S
**McGill et al. (2007)**		x	-	-	-	-	-	-	M	N/A	S
**Rose et al. (2014)**		x	-	-	-	-	-	-	Pos	N/A	S
**Tierney et al. (2007)**		x	-	-	x	-	-	-	N	N/A	S
**van Oorsouw et al. (2010)**		x	-	-	x	-	-	-	Pos	N/A	S
*Knowledge and Implementation*												
**Gormley et al. (2019a)**	*K*	x	-	x	x	x	-	-	M	N/A	P
	*I*	x	-	x	x	x	-	-	NI		
**MacDonald et al. (2018)**	*K*	x	-	-	-	-	-	-	M	M	S
	*I*	⇓	-	-	x	x	-	-	NI		
**Macurik et al. (2008)**	*K*	x	-	-	-	-	-	-	NI	N/A	Con
	*I*	x							NI	N/A	
*Implementation*											
**Crates and Spicer (2012)**		x	-	x	-	x	-	-	NI	Pos	S
**Courtemanche et al. (2014)**	Phase 1	x	-	x	x	x	-	x	Pos	M	Con
	Phase 2	⇓	-	-	-	x	x	x	Pos	Pos	
	Phase 3	⇓	-	-	-	x	x	x	Pos	Pos	
	Phase 4	⇓	-	-	-	x	x	x	Pos	M	
**Jarmolowicz et al. (2008)**	Technical Group (TG)	x	-	-	x	-	-	-	N **(C)**	N/A	P
	Non- TG	x	-	-	x	-	-	-	Pos **(I)**	N/A	
**McClean and Grey (2012)**		x	-	-	-	x	-	-	NI	Pos	S
**Shore et al.(1995)**	Baseline	x	-	VM	-		-	-	N	M	P
		x	-		-	x	-	-	Pos	M	

Note* *C = control, Con = conclusive, I = implementation, I = intervention, K = knowledge, M = mixed, N/A = not applicable, N = negative, NI = not interpretable, Pos = positive, P = preponderant, S = suggestive, VM = video modelling, ⇓ = Intervention effects carried over.*

### Participants

The 18 studies included 1352 participants, and 10 of the 18 studies (55.6%) reported age characteristics, with ages ranging from 19 to 63 years. Eleven of the 18 studies (61.1%) included information on gender characteristics (1027 participants) 76.7% (n = 788) were female and 23.2% (n = 238) were male. Participants included staff that supported individuals with intellectual disabilities (n = 6, 33.3%), intellectual and developmental disabilities, who engaged in challenging behaviour (CB) (n = 5, 27.8%), severe disabilities (Macurik et al., 2008) developmental disabilities and psychiatric disorders (Berryman, et al., 1994) and varying diagnosis who engaged in self-injurious behaviour (Courtemanche et al., 2014; Shore et al., 1995). Participants also included nursing assistants, nurses and managers in health care services (Lowe et al., 2007), students completing a diploma (McGill et al., 2007), and staff in disability services (Crates and Spicer, 2012; Tierney et al., 2007). Eight of the 18 studies (44.4%) reported information on participant’s prior level of PBS knowledge. Experience of participants varied across studies. Berryman et al. (1994) and Hardesty et al. (2014) reported that participants received orientation training related to behaviour management/behavioural principles and strategies, upon commencing their job. In addition, Berryman et al. (1994) described that participants received 2 days of training yearly and asked participants to rate how familiar they were with content of training, similarly in Gormley et al. (2019a) participants were asked if they had received training on the specific content taught. In contract, other studies required that participants not to have participated in a comparable training regarding the management of CB for at least 2 years (van Oorsouw et al., 2010) and to not have attended a CB training within the last 6 months (Rose et al., 2014). Across other studies participants had limited experience with CB training (not specified) (McGill et al., 2007), and no prior training (Branch et al., 2018). Jarmolowicz et al. (2008), compared experienced versus inexperienced participants. Experienced participants had at least 1 years’ experience of graduate study in Applied Behaviour Analysis (ABA) whereas inexperienced participants had no formal training.

### Design

Five studies (27.8%) had experimental research designs, including single subject research designs (*n =* 2, 11.1%), between group designs with random allocation (*n =* 4, 22.2%). Twelve studies had quasi-experimental designs, including within subject designs (*n =* 4, 22.2%), repeated measures design (*n =* 2, 11.1%), and mixed research design (which included both within subject and between subject’s design, without random allocation *n =* 6, 33.3%).

### Measures

Thirteen studies (72.2%) took measures of knowledge. Fourteen different measures were used across these studies comprising of six standardized measures including the Test of Knowledge (*n =* 1, 7.7%), Causal Attributions for Challenging Behaviour Scale (*n* = 1, 7.7%), Challenging Behaviour Representation Questionnaire (*n =* 1, 7.7%), modified Self-Injury Questionnaire (*n =* 2, 15.3%), Controllability Beliefs Scale (*n =* 1, 7.7%), and the Challenging Behaviour Attribution Scale (CHABA, *n =* 3, 23%), seven measures were not standardized and included written/multiple choice quizzes related to the content taught, and one study used verbal responses to a quiz. Gormley et al. (2019a), MacDonald et al., (2018) and Macurik et al. (2008), took measures on both knowledge and implementation. Implementation measures were reported in 8 studies (44.4%), with the use of measures of fidelity including Periodic Service Review (*n =* 3, 37. 5%), correct implementation (*n =* 4, 50%) and performance score (*n =* 1, 12.5%). Other measures not related to knowledge or implementation included measures on attitude towards individuals with disabilities (*n =* 1, 5.6%), evidenced based practice (*n =* 1, 5.3%), perceptions of self-efficacy (*n =* 2, 11.1%), confidence (*n =* 1, 5.6%), supervisory support (*n =* 2, 11.1%), and emotional reactions to CB (*n =* 3, 16.7%). Please see Design and Measures section in Table 1 for description of these additional measures.

### Training content

Of the studies which focused on knowledge (*n =* 13, 72.2%), Branch et al. (2018) was the only study in which content was on a specific BSP. All other studies delivered training on positive behavioural interventions (*n* = 1, 7.7%), approaches to supporting individuals with CB (*n* = 1, 7.7%), an introduction to ABA (*n =* 2, 15.3%), role of environment for CB (*n* = 1, 7.7%), specific behavioural interventions (*n* = 1, 7.7%), principles of PBS (*n =* 3, 23%), behavioural principles (*n =* 3, 23%), challenging behaviour courses (*n =* 3, 23%). Of the studies which further outlined content, functional analysis (*n =* 2, 15.3%), functional communication training (*n =* 4, 28.6%), observation (*n =* 2, 15.3%), understanding CB (n = 5, 38.5%), and behaviour support planning (*n* = 1, 7.7%), was delivered as part of the training content. Of the studies which focused solely on implementation (*n =* 8, 44.4%), training content focused on training specific behavioural interventions as part of BSPs (*n =* 3, 37.5%), function-based interventions (*n =* 2, 25%), and training participants how to conduct functional assessments, develop BSPs and implement them (*n =* 2, 25%). Across these eight studies, 87.5%, (n = 7) included differential reinforcement, 75%, (n = 6) functional communication training and proactive strategies, 100%, (n = 8) reactive strategies, 12.5%, (n = 1) included punishment strategies (loss of tokens, response blocking) and 25%, (n = 2) included extinction.

### Format of training

Format was not directly reported for five studies (27.8%) but was extracted from the procedure description. From the studies which took measures of participant knowledge (*n =* 13, 72.2%), 76.9% (n = 10) of studies had a group format, Campbell and Hogg (2008) used an individual format, as it was a distance-learning course and McGill et al. (2007) used a combined group and individual format. Fifty per cent (n = 4) of implementation studies used a group format, 25%, (n = 2) used an individual format and 25%, (n = 2) a combined format.

### Duration of training

#### Studies which measured knowledge

Training duration varied across studies which solely focused on knowledge, from 2-hour workshops (Hardesty et al., 2014), 1 day workshops (Berryman, et al., 1994; Dowey et al., 2007; Rose et al., 2014), 3 day workshops (Tierney et al., 2007), 4 hour workshops, practice across 4 weeks, support once a week (Branch et al., 2018), 24.5 hours across 7 workshops (van Oorsouw et al., 2010) to 57 days of workshops spread across 2 years (McGill et al., 2007). Distance learning, with each module to be complete within a 3-month period (Campbell and Hogg, 2008) and 80 hours direct teaching across 10 consecutive days (32 h home-based study leave, 40 h work-based study time and were expected to contribute 28 h of their personal time) (Lowe et al., 2007).

#### Studies which measured both knowledge and implementation

Duration also varied across studies which assessed both implementation and knowledge (*n =* 3, 16.7%). Macurik et al. (2008) found that live training averaged 23, 31 and 46 minutes, while video training averaged 10, 21 and 35 minutes. Gormley et al.’s (2019a) training duration was 20 hours across 3 consecutive days, while MacDonald et al. (2018) was 10 days.

#### Studies which measured implementation

From the implementation studies, three studies (37.5%) did not report duration of training but provided information on the criterion required to progress. Of the studies which did report this variable, duration of training ranged from 10 full days of training and coaching across 9 months (McClean and Grey, 2012), to 13 days, including longitudinal practicum spread over a 9-month period (Crates and Spicer, 2012).

### Training components

#### Across all studies

Description was used across all studies, in conjunction with discussion (*n =* 8, 44.4%), including individual activities (*n =* 1, 5.6%), group activities (*n =* 4, 22.2%), group and individual activities (*n =* 2, 11.1%), technical versus non-technical language (*n =* 1, 5.6%), case studies and up to date research findings (*n =* 1, 5.6%), practical exercises, (*n =* 1, 5.6%), and video tapes (*n =* 1, 5.6%), Only Dowey et al. (2007) specified what the group activities entailed. See Table 2 for a summary of training components.

#### Studies which measured knowledge

Five studies (38.5%) used description as the sole method of training (in conjunction with small and group activities). Three studies (23.1%) utilized both description and role-play (Dowey et al., 2007; Tierney et al., 2007; Van Oorsouw et al., 2010). One study (7.7%) used description and fluency (Branch et al., 2018) and Hardesty et al., (2014) utilized description and feedback, comparing individual feedback to group feedback through the use of response cards.

#### Studies which measured both knowledge and implementation

Macurik et al. (2008) utilized description as the training method. Gormley et al. (2019a) utilized behavioural skills training: description, role-play, modelling and feedback, while MacDonald et al. (2018) utilized description and feedback.

#### Studies which measured implementation

Seventy-five per cent of studies (n = 6) that measured implementation (*n =* 8) utilized feedback, 50% (n = 4) used modelling and role-play. Two studies (25%) utilized the training package of description, modelling, and feedback (Crates and Spicer, 2012; Shore et al., 1995). Shore et al. (1995) compared description and video modelling to description, video modelling and *in vivo* feedback. McClean and Grey (2012) utilized description and feedback. Jarmolowicz et al. (2008) utilized description (technical language description versus non-technical description) and role-play. Courtmanche et al. (2014) compared a training package of description, modelling, role-play, feedback, escape contingency, monitory incentive across four different conditions.

### Outcomes and certainty of evidence

#### Studies which measured knowledge

Six out of 13 studies (46.2%) that measured knowledge (three standardized measures and four unstandardized) had positive outcomes (Berryman et al., 1994; Branch et al., 2018; Dowey et al., 2007; Hardesty et al., 2014; Rose et al., 2014; van Oorsouw et al., 2010). Five studies (38.5%) had mixed outcomes with two standardized measures and two with both standardized and unstandardized (Campbell and Hogg, 2008; Gormley et al., 2019a; Lowe et al., 2007; MacDonald et al., 2018; McGill et al., 2007). Tierney et al. (2007) had negative outcomes (standardized measure). Macurik et al., (2008) results were not interpretable. Of the studies that had positive outcomes, Berryman et al., (1994) had the strongest certainty of evidence: preponderant due to not including a measure of treatment fidelity. This study utilized the sole intervention component of description. All other studies that had positive outcomes had suggestive levels of certainty. Of the studies that had mixed outcomes, Gormley et al. (2019a) had the strongest certainty of evidence: preponderant due to no baseline data being measured for skills. However, a positive outcome was identified for knowledge from a training package of description, modelling, role-play and feedback (Gormley et al., 2019a). All other studies that had mixed outcomes had suggestive levels of certainty. For MacDonald et al. (2018) an increase in knowledge on the specific PBS test, but not the CHABA, was identified for managers. However, there were no significant increases for frontline staff on either measure. Tierney et al. (2007) which had negative outcomes had a suggestive level of certainty and combined description and role-play. MacDonald et al. (2018) was the only knowledge study which took client measures with mixed outcomes.

#### Studies which measured implementation

Three out of eight studies that took measures on implementation had positive outcomes (Courtemanche et al, 2014; Jarmolowicz et al., 2008; Shore et al., 1995). Five studies (62.5%) were not interpretable (Crates and Spicer, 2012; Gormley et al., 2019a; MacDonald et al., 2018; Macurik et al., 2008; McClean and Grey, 2012). Of the studies that had positive outcomes, Courtemanche et al. (2014) had the highest level of certainty: conclusive. Courtmanche et al. (2014) compared a training package of description, modelling, role-play, feedback, escape contingency, monitory incentive across four different conditions. The use of all six components had positive outcomes for participants. Jarmolowicz et al. (2008) and Shore et al. (1995) had the next strongest level of certainty: preponderant due to treatment fidelity not being reported. Jarmolowicz et al. (2008) utilized description and role-play. They compared a technical language description (negative outcomes) which they found to be less effective than a non-technical description (positive outcomes). Shore et al. (1995), evaluated a training package consisting of description, modelling, and feedback. Description and video modelling were compared to description, video modelling (carried over from previous condition) and *in vivo*. Of the studies that had mixed outcomes, Gormley et al. (2019a) was preponderant due to no baseline data being measured for skills utilizing behavioural skills training.

Five out of eight studies which evaluated participant implementation, took measures on client outcomes. Two of these studies (Crates and Spicer 2012; McClean and Grey, 2012) had positive outcomes for clients. Courtemanche et al. (2014), MacDonald et al. (2018), and Shore et al. (1995) had mixed outcomes for clients.

### Maintenance and generalization

Eleven studies (61.1%), (nine knowledge, two implementation) took measures of maintenance, ranging from 2 weeks (Branch et al., 2018; Hardesty et al., 2014) to 1 year (Lowe et al., 2007). Berryman et al. (1994) found effects to be maintained at 9-month follow-up, Rose et al. (2014) and van Oorsouw et al. (2010) at 2 months. Branch et al. (2018) and Hardesty et al. (2014) found effects to be maintained for the fluency group and response card groups at 2-weeks. Lowe et al. (2007) found that at 1-year follow-up, CHABA scores had generally returned to baseline levels but for the sub-group which completed the knowledge questionnaire, there was an increase in knowledge score at follow-up. Campbell and Hogg (2008) found no statistical difference between groups at the 3-month post-test. There were no measures of generalization taken for knowledge. Generalization of skills learned in training to working with the client was taken for 50% (n = 4) of implementation studies.

### Social validity

Three knowledge studies (23.1%) measured social validity of training delivered (Hardesty et al., 2014; Lowe et al., 2007; van Oorsouw et al., 2010). Participants in the response card group in Hardesty et al. (2014) and Lowe et al.’s (2007) study rated training positively. van Oorsouw et al. (2010) participants rated the training from acceptable to good. Four implementation studies (50%) measured social validity of the training carried out (Courtmanche et al., 2014; Crates and Spicer, 2012; Gormley et al., 2019a; Macurik et al., 2008), with all trainings being rated positively. Jarmolowicz et al. (2008) and McClean and Grey, (2012) measured social validity of the procedures trained rather than training method.

## Discussion

The current review aimed to evaluate the most effective staff training methods used to increase knowledge and implementation of BSPs among staff supporting individuals with intellectual and developmental disabilities in residential or day settings. In addition, the review aimed to identify the components responsible for training efficacy. A total of 18 studies were included in the review, 13 of which measured knowledge and 8 measured implementation, while 3 articles measured both dependent variables. Descriptive analysis of the included studies led to the following conclusions.

Description, despite being the most commonly used training component, when used in isolation did not consistently result in increasing staff knowledge of PBS. Of the two studies that had positive outcomes (Berryman, et al., 1994; Rose et al., 2013), certainty of evidence was at the preponderant and suggestive levels, thus prohibiting support for the use of description in isolation to increase staff knowledge of PBS. Description when used in combination with other strategies resulted in acquisition of knowledge, in particular when description was combined with fluency training or group feedback. However, description combined with role-play was found to have mixed results (effective within two studies but ineffective in another). Descriptions from two of the three studies outlined that role-play’s were used to demonstrate specified physical intervention skills (van Oorsouw et al., 2010) and personal safety techniques (Tierney et al., 2007). Therefore, it is not clear if the role-play’s targeted knowledge specifically. Gormley et al. (2019a) was found to have positive outcomes for knowledge following the use of a training package consisting of description, rehearsal and feedback. Small and group activities were often used in conjunction with description, but sufficient detail was not provided, therefore, it was not possible to determine whether modelling, role-play, or other strategies were incorporated within these.

Description, modelling, feedback and role-play were the most commonly used training components in different combinations across all eight implementation studies. Description was effective at increasing implementation when used in combination with: (i) feedback; (ii) role-play; and (iii) modelling, role-play, feedback, monitory incentive and escape contingency (Courtmanche et al., 2014). Shore et al. (1995) found description and video modelling alone weren’t successful, requiring the addition of *in vivo* feedback. Current literature evaluating component analysis of BST have utilized single subject research design methods and are not specific to training frontline staff to implement BSPs (Davis, Thomson and Connolly, 2019).

As well as variance in the combination of components included in the training packages there were differences across the type of components. For example, modelling included *in vivo* modelling with staff members (Courtmanche et al., 2014) and video modelling which included clients (Gormley et al., 2019a; Shore et al., 1995). Similarly, the types and quantity of feedback given varied including written, verbal, *in vivo*, delayed, and immediate. Within the current review, *in vivo* modelling was effective in a combination package, while video modelling when used in isolation with description was not effective. These findings further emphasize the need for component analysis of training strategies in order to identify the most effective and efficient components.

One factor which may warrant further consideration during training is the language used. Jarmolowicz et al. (2008) reported that the use of non-technical description and role-play resulted in greater staff implementation compared to the use of a technical description and role-play (Jarmolowicz et al., 2008). As the majority of training is designed for frontline staff, the use of non-technical language should be considered when preparing training materials as well as when developing BSPs. The use of a monetary incentive was found to be an effective component within a training package by one study. However, the use of this component within a training package requires careful consideration as it may not be feasible for organizations to support this component in the long term.

Of the studies which examined staff knowledge, client outcomes were not measured. Therefore, the impact of change in knowledge upon the individuals supports is not known. In contrast, five of the eight studies which measured implementation reported client outcomes. All five studies reported a reduction in CB (Courtemanche et al., 2014; Crates and Spicer, 2012; MacDonald et al., 2018; McClean and Grey, 2012; Shore et al., 1995), and one study measured quality of life (MacDonald et al., 2018), with no significant change reported. Therefore, further research is needed to investigate the impact of BSPs on the quality of life for the individual supported (MacDonald and McGill, 2013). An observation from these findings is that it may be challenging to assess QOL following briefer workshops, whereas this may be more feasible in longitudinal trainings such as in McClean and Grey, (2012). Alternatively, it may be worth gathering data on whether the clients acquired adaptive behaviours as a result of the staff training, to assess if the training was having a direct impact on socially significant behaviours for the clients.

The current review builds on MacDonald and McGill (2013) by including a thorough descriptive analysis of training components and related outcomes. Similar to the findings of their review, there was variation in format, duration, content, measures used across studies reported, which must be taken into account when interpreting findings. Within the current review, seven studies included frontline staff managers in the training, for five of these there was no differentiation between supervisor outcomes and frontline staff outcomes. Shore et al. (1995) trained the managers to train the frontline staff and in MacDonald et al. (2018) study, the managers were the primary participants that oversaw the implementation of the BSPs after receiving training. Shore et al. (1995) demonstrated that the managers were effective in training the frontline staff to implement the BSPs. These findings also indicate that key personnel (in this instance managers) could also be successfully trained (Shore et al., 1995). This is a consideration for organizations in terms of cost effectiveness. MacDonald et al. (2018) utilized managers to observe and provide feedback to staff on their implementation, while data was not taken on the frontline staff’s implementation it is not possible to interpret its impact. However, having mangers on-site who are skilled in this way may have other benefits of providing ongoing feedback and monitoring. Therefore, further research should evaluate pyramidal training.

To enhance the current knowledge and practice base, it is essential to assess and identify active components of current training models. Future research should also consider cost effectiveness in terms of brief skills-based training (e.g. Gormley et al., 2019a) versus longitudinal training (McClean and Grey, 2012) in terms of best outcomes for acquiring skills, knowledge, client QOL, generalization, maintenance, social validity and what is ecologically valid for organizations. However, prior to evaluations of person focused, brief and pyramidal training, which would impact on development of overall training approaches, it is crucial to identify the active training components (e.g. feedback) necessary for frontline staff to acquire implementation skills which could then be incorporated within these training models.

Within the current review a wide variety of measures (both standardized and unstandardized) were used to measure change in participants knowledge, attributions, behavioural explanations and implementation of specific behavioural interventions. Standardized measures had mixed outcomes whereas unstandardized measures all had positive outcomes. Only two studies used both standardized and unstandardized measures. In order to evaluate the full impact of training it may be important to include both standardized and training specific measures to investigate both changes in attributions/understanding of CB and knowledge of taught procedures. It also raises the question of whether there is a need for refresher courses on the theory around functions of behaviour, whereas knowledge on interventions that are being implemented on a daily basis may be more likely to be maintained.

Across all studies (both implementation and knowledge), there was a lack of information provided on participants previous experience. The information provided focused on how long participants had been working with individuals with intellectual and developmental disabilities or in the particular setting, but not specifically related to PBS training/experience.

In terms of study designs, is was not possible to interpret the findings with respect to implementation for larger scale group designs (Gormley et al., 2019a; MacDonald et al., 2018; Macurik et al., 2008; McGill and Murphy, 2018), as no baseline measures were taken. These studies incorporated training staff on the principals of PBS/ABA and on specific elements of BSPs. The challenges around obtaining baseline measures of implementation, in large group designs, in residential or day-care settings must be acknowledged due to reactivity, time constraints and the resources required. Simulated role-play baseline assessments which are more often used in educational settings (Shapiro and Kazemi, 2017) may be a viable alternative, while incorporating an on-site observation for a select intervention. If the most commonly recommended behavioural interventions of BSPs are identified, they could be used as the target interventions for baseline assessment and incorporated as the training content. It will be likely that these interventions will be implemented more frequently and reflect an accurate representation for baseline skills which can then be assessed post training. This may also serve to enhance the social validity of training for staff. In an instance that the BSPs are already developed, specific examples from active BSPs may further enhance social validity.

Implementation studies which measured generalization focused on generalization from the training environment to working with the client. A consideration for Jarmolowicz et al. (2008), was staff’s performance with a confederate (not a client), which highlights the need to determine if those skills would have generalized to working with the individuals the staff support. Across studies, maintenance was assessed more frequently in knowledge-based studies. However, it is recommended that further evaluations of knowledge include longer term follow-up, such as 9 months (Berrryman et al., 1994) and 1 year (Lowe et al., 2007). In addition, future research could examine maintenance across attributions about CB versus acquired knowledge of PBS, and specific behavioural interventions. Finally, within the current review 12 studies had suggestive certainty of evidence, which indicates that there is a lack of fidelity measures taken. Therefore, the quality of the training provided to the staff is not clear.

Future research in the area of staff training could be enhanced by specifying participants previous experience, as to date, the impact of previous PBS training, knowledge and experience is unknown. In order to control for previous experience as an extraneous variable it is necessary for studies to account for this factor. Similarly, clear descriptions of ‘additional activities’ are necessary to enhance rigor, as well as the use of experimental designs to evaluate the effects of staff training on knowledge and implementation of BSPs. While there were many strengths to this review, limiting the participants to staff that provide care for individuals with intellectual and developmental disabilities in residential and day settings may have resulted in missed studies examining BSP implementation in school settings, however other reviews have included these settings.

This review further supports Brady et al. (2019) findings for the need to evaluate different components of current training models and builds on it by specifically focusing on BSPs and residential/day settings. It also extends on the work of Brady et al. (2019) by highlighting in detail methodological factors necessary to achieve high quality research that would enable evidence-based practice to be identified.

In conclusion, to teach knowledge of PBS to staff, the training component description alone is not consistently effective and should be used in combination with other training strategies. In intellectual and developmental disabilities services (residential and day settings), the primary goal is the implementation of BSPs, which is supported by staff knowledge of both CB and of the behavioural interventions. Targeting both of these areas of knowledge is important. For staff to acquire the necessary skills to consistently implement the BSPs, description combined with feedback or role-play are effective packages, as are more complex training packages that include more than four components. However, direct evaluation using experimental rigorous designs to identify which of these components are efficacious, necessary, efficient, feasible and cost-effective, should be conducted to identify evidenced based practice when training staff to implement BSPs.
